# The Association Between Estrogen-Containing Oral Contraceptive Pills and Hypothyroidism

**DOI:** 10.1155/ije/5978558

**Published:** 2025-03-18

**Authors:** Lama Alkahlout, Shahd Hamran, Nour Darwish, Yara Dweidri, Giridhara R. Babu, Rafif Mahmood Al Saady

**Affiliations:** ^1^College of Medicine, QU Health, Qatar University, Doha, Qatar; ^2^Department of Population Medicine, College of Medicine, QU Health, Qatar University, Doha, Qatar; ^3^Department of Basic Medical Sciences, College of Medicine, QU Health, Qatar University, Doha, Qatar

**Keywords:** cross-sectional study, estrogen, hypothyroidism, oral contraceptive pills (OCP), sex hormone-binding globulin (SHBG)

## Abstract

**Background:** Hypothyroidism is an endocrine disorder that affects 10 times more females than males, with substantial health impacts. The role of estrogen-containing oral contraceptives (OCPs) in influencing thyroid function remains relatively underexplored. This study investigated the association between estrogen-containing OCP use and hypothyroidism in the female population in Qatar.

**Methods:** We utilized data from the Qatar Biobank to conduct a cross-sectional study of 1001 female participants with a current or previous history of estrogen-containing OCP use. The thyroid function of the participants was evaluated via thyroid function test parameters (thyroid stimulating hormone (TSH) and free tetraiodothyronine (fT4)) as outcomes, and sex hormone-binding globulin (SHBG) levels as a proxy indicator of OCP use. We adjusted for confounders such as age, ethnicity, and type 2 diabetes mellitus status via multivariable logistic regression to determine the odds of hypothyroidism associated with SHBG levels.

**Results:** Among the 1001 participants, 34 (3.4%) met the diagnostic criteria for hypothyroidism. Multinomial logistic regression revealed no increased odds of hypothyroidism at the 90th percentile cutoff for SHBG levels (OR 1.00, 95% CI 0.29–3.50). However, at the 95th percentile of SHBG values, there was 46% higher odds of hypothyroidism (OR 1.46, 95% CI 0.33–6.54) and an over tenfold increase at the 99th percentile (OR 10.07, 95% CI 1.94–52.45, *p*=0.006). Other variables, such as age, ethnicity, and diabetes status, were not associated with SHBG levels. Non-Qatari Arabs had higher odds of hypothyroidism compared with that of Qataris (OR 8.06, 95% CI 0.84–77.54, *p* value 0.071).

**Conclusion:** This study offers evidence that SHBG levels can be used to indicate estrogen-containing OCP use. Furthermore, higher SHBG levels are associated with higher odds of hypothyroidism among OCP users, and the odds of hypothyroidism vary across different ethnicities. These findings highlight the need for further research to explore the mechanisms linking OCP use to thyroid dysfunction and to investigate other potential risk factors.

## 1. Introduction

Hypothyroidism is a prevalent endocrine disorder with varying global incidence rates. In Europe, an estimated 5% of the population is affected [1], whereas the NHANES III study in the USA reported a prevalence of 4.6% [2]. In the Arab region, hypothyroidism rates are 8.8% in the UAE, 3.1% in Jordan, and 5.3% in parts of Saudi Arabia [3–5]. Approximately 5% of the population in Qatar, predominantly women, are affected, mirroring trends observed throughout the Middle East [6].

Primary hypothyroidism, which accounts for more than 99% of cases, results from inadequate production of thyroid hormones in the thyroid gland [1]. This condition can be classified as either clinical or subclinical, with clinical hypothyroidism characterized by elevated thyroid stimulating hormone (TSH) and low free thyroxine (T4) levels. In contrast, subclinical hypothyroidism presents with elevated TSH and normal free T4 levels [7]. The regulation of thyroid hormone production involves the hypothalamic–pituitary–thyroid (HPT) axis, where the hypothalamus releases thyroid-releasing hormone (TRH), prompting the anterior pituitary gland to secrete TSH, which stimulates the thyroid gland to produce triiodothyronine (T3) and T4 [8, 9]. Once synthesized, these hormones are predominantly transported in the bloodstream and are bound to transport proteins such as thyroxine-binding globulin (TBG), transthyretin, and albumin [10]. Globally, iodine deficiency is a leading cause of hypothyroidism, particularly in regions with limited iodine intake. However, in iodine-sufficient areas such as Qatar, autoimmune thyroiditis (Hashimoto's disease) is the most common cause [7, 11]. Emerging evidence suggests that other factors, such as oral contraceptive pills (OCPs), may also influence thyroid function, mainly because of their estrogen content [12].

Estrogen, an essential female hormone, regulates the reproductive cycle and protects against some conditions, such as osteoporosis. It is also administered exogenously in OCPs containing synthetic estrogens such as mestranol and ethinyl estradiol [13]. Given the potential long-term health risks of OCP use, such as myocardial infarction and venous thrombosis [14, 15], exploring their impact on thyroid hormone levels is essential. While the mechanisms linking estrogen and thyroid hormone regulation are not fully understood, research suggests that estrogen can increase TBG sialyation, reduce renal clearance and increase serum concentrations [12]. Additionally, estrogen administration through OCPs has been shown to increase sex hormone-binding globulin (SHBG) levels, which may further influence thyroid hormone levels [16].

SHBG is an indicator of estrogen-containing OCP use due to its sensitivity to estrogen levels, as documented by several studies. A study in 2014 revealed that combined OCPs significantly increased SHBG levels, with a smaller increase noted in those using second-generation progestins or lower estrogen doses [17]. Another study in 2006 focusing on premenopausal women with sexual dysfunction reported that SHBG levels were up to four times higher in OCP users than in those who had never used them and that SHBG levels remained elevated even after discontinuation [16]. SHBG levels due to OCP use can increase by 80% to 300% [16]. The exact mechanism by which elevated estrogen increases SHBG remains unclear. Nevertheless, it is hypothesized that estrogen induces SHBG synthesis, as evidenced by the higher SHBG levels in OCP users and during ovulation [18]. Estrogen may also increase SHBG levels by increasing its glycosylation, which reduces clearance rates, a mechanism similarly proposed for the increased levels of TBG observed with elevated estrogen [19].

This study explores the relationship between estrogen-containing OCP use and thyroid hormone levels in a female population in Qatar. Using data from the Qatar Biobank (QBB), we aimed to understand how OCPs may affect thyroid function. This research could provide valuable insights into the long-term health implications of OCP use and inform clinical guidelines for managing thyroid health in women who use these contraceptives.

## 2. Materials and Methods

### 2.1. Study Design

This cross-sectional study utilized data from the QBB database. The QBB is a research initiative that collects data from the Qatari population and long-term residents for scientific research, with the aim of developing public health outcomes tailored to the local population. Due to their data collection method and available data sets for research use, certain exposures, such as dosage and duration of OCP use, are unattainable. This limits our analysis and prediction model.

### 2.2. Eligibility

Women with a current or previous history of estrogen-containing OCPs were enrolled in this study. They were then evaluated for thyroid gland function via the thyroid function test parameters TSH and free T4. SHBG was used as a proxy indicator of OCP use. Demographic information such as age, nationality, the basal metabolic index (BMI), smoking status, and chronic medical conditions such as diabetes mellitus (DM) were obtained from the QBB database. The inclusion criteria in this study were being 18 years of age and above, using or having used estrogen-containing oral contraceptives, having thyroid function test results, and having data regarding their SHBG level. Women were excluded if they were on thyroid hormone medications such as levothyroxine, were on medications that can interfere with thyroid gland function (such as lithium or amiodarone), had a known diagnosis of thyroid cancer, were pregnant or had missing data.

### 2.3. Outcome Measures

Our primary outcome was the presence of overt hypothyroidism as assessed by thyroid function tests.

### 2.4. Thyroid Function Test Analysis

The parameters used were TSH levels and free tetraiodothyronine (fT4) levels. We identified the participants with overt hypothyroidism based on the normal laboratory values in Qatar's laboratories: low T4 of < 10.3 mcg/dL and TSH > 4 mU/L. These criteria align with the widely accepted diagnostic framework for overt hypothyroidism [20]. Free T3 (fT3) was not included in the analysis because it is not a standard diagnostic criterion for hypothyroidism and is typically the last thyroid function test to become abnormal in such cases.

### 2.5. Covariates

The confounders were identified by reviewing the relevant literature to determine the factors influencing the use of OCPs or thyroid function. A directed acyclic graph (DAG) was then used to pinpoint which factors acted as confounders in our study (Supporting File (available here)). The confounders identified and adjusted for were age [21, 22], ethnicity [23, 24], and type 2 DM (T2DM) status [25, 26].

### 2.6. Statistical Analysis

Frequencies and proportions were reported for categorical variables, whereas bar graphs and pie charts were used to present data where appropriate. The chi-square test, Fischer's exact test (small frequencies), and McNamar's chi-square test (paired data) were used to compare categories. All continuous data were tested for normality via histograms. Means and standard deviations are presented for normally distributed variables, whereas medians and IQRs are reported for variables that do not follow a normal distribution. SHBG was grouped into quartiles, and we assessed different percentiles (90%, 95%, 99%). *T* tests and ANOVA (for more than two groups) were used to compare continuous outcomes between groups if the data were normally distributed. For data that were not normally distributed, comparisons were performed via the Mann–Whitney test, the Wilcoxon rank-sum test (2 independent groups), the Wilcoxon rank test (paired data), or the Kruskal–Wallis test for more than two groups. Multivariate logistic regression was used to synthesize the odds of developing the primary outcome. We performed multinominal logistic regression analysis examining the associations between hypothyroidism and several predictor variables using the 90^th^, 95^th^, and 99^th^ percentiles (140.5, 202.55, and 378 nmol/L, respectively) as our cutoff values for SHBG levels. We performed a link test for the multinomial regression model for the odds of hypothyroidism in participants with SHBG levels higher than the 99^th^ percentile while adjusting for age, ethnicity, and diabetes status. Odds ratios (ORs), 95% CIs and exact *p* values are reported. We conducted a multinomial logistic regression analysis for the associations between hypothyroidism and several predictor variables, including SHBG quartiles, age, and diabetes status. All the statistical analyses were performed via Stata 18 (College Station, TX, USA).

## 3. Results

### 3.1. Sample Characteristics

We included a total of 1001 participants, which included 967 individuals who did not have hypothyroidism and 34 individuals who met the diagnostic criteria for hypothyroidism. The age distribution was similar in both groups, with a median age of 44 years in the no hypothyroidism group and 45 years in the hypothyroidism group. Among the participants with no hypothyroidism, 26.9% of the women had T2DM, whereas 73.1% of the women did not have T2DM. Among women who had hypothyroidism, 29.4% had T2DM, whereas 70.6% did not have T2DM. With respect to smoking status, only 2.5% of the women with no hypothyroidism were smokers, whereas the remaining women with no hypothyroidism were nonsmokers. For women with hypothyroidism, none of them were smokers, while one woman's smoking status was not documented. A total of 94.4% of women without hypothyroidism were Qatari, 5.2% were non-Qatari Arabs, and 0.4% were from other nationalities. Among the women with hypothyroidism, 87.9% were Qatari, 9.1% were non-Qatari Arabs, and 3% were from other nationalities. Most of our sample had a BMI > 30 in both groups.  Table 1 suggests that there were no differences between the baseline characteristics (age, T2DM status, and smoking status) of our study sample (Table 1).

Table 2 provides an overview of the characteristics observed within our study sample, categorized according to SHBG levels. Within our study sample, 11 participants had an SHBG level ≥ 99^th^ percentile. The participants tended to be significantly younger, with a median age of 34 years compared with 44 years for the 990 women with SHBG levels < the 99^th^ percentile. The distribution of diabetes between the two SHBG groups was not different (*p*=0.18), and there was a noticeable trend toward higher rates of diabetes among women with lower SHBG levels, suggesting a potential link worthy of further investigation. The proportion of women with hypothyroidism was significantly greater in the > 99th percentile group, with 27.3% (three women) affected, than in the < 99th percentile group, with only 3.1% (31 women). The majority of patients in the < 99^th^ percentile group had no hypothyroidism. Finally, most of our participants were nonsmokers, regardless of their SHBG levels (Table 2).

The results of the multinomial logistic regression analysis for the associations between hypothyroidism and SHBG quartiles adjusted for age, diabetes status, and ethnicity are provided in Table 3, in addition to the different predictors for hypothyroidism. The odds of hypothyroidism were 31% greater in people with SHBG levels within the second quartile than in those with SHBG levels within the first quartile (OR 1.31, 95% CI 0.46–3.72, *p* value = 0.612) and increased in the third and fourth quartiles (OR 1.5, 95% CI 0.54–4.22, and OR 1.65 95% CI 0.57–4.78, respectively), with poor evidence against the null hypothesis. Upon assessment of the different predictors of the odds of hypothyroidism, no associations were detected for age or diabetes status. However, being of Arab, non-Qatari ethnicity seemed to increase the odds of hypothyroidism compared with that of Qataris by more than eight times, with moderate evidence (*p* value = 0.071). Furthermore, smoking was omitted from the model, as all patients with hypothyroidism were nonsmokers. In conclusion, increases in SHBG levels were associated with hypothyroidism in this model.

The results revealed no increase in the odds of hypothyroidism at the 90^th^ percentile cutoff (OR 1.00, 95% CI 0.29–3.50), but there was an incremental trend toward a 46% increase in the 95^th^ percentile and more than eight times greater odds of hypothyroidism in individuals with SHBG levels ≥ 99^th^ percentile than in those with SHBG levels < 99^th^ percentile (OR 1.46, 95% CI 0.33–6.54; OR 10.07, 95% CI 1.94–52.45, *p* value = 0.006). Other variables, such as age, ethnicity, and diabetes status, were not associated with hypothyroidism in this model (Table 4).

The *p* value for hat squared was 0.622 for the odds of hypothyroidism, indicating that the model specification was adequate. The only coefficient for hat was significant, with a value of 1.94 for the odds of hypothyroidism. This suggests that some predictor variables may not have been adjusted for in the models.

## 4. Discussion

In this cross-sectional study of 1001 female participants with a current or previous history of estrogen-containing OCP use, we found a 10-fold increase in the odds of hypothyroidism in participants with SHBG levels above the 99^th^ percentile. Our findings also indicate that the odds of hypothyroidism increase with each successive increase in the SHBG quartile, with poor evidence against the null. The incremental increases in the odds of hypothyroidism with SHBG quartiles and increased SHBG percentile cutoffs suggest a positive association between these variables.

Our findings align with those of previous studies conducted in the USA between 2007 and 2012, which similarly reported higher odds of hypothyroidism among women who had ever used OCPs (OR 1.245, 95% CI 1.04–1.49). The odds were even greater among women who had used OCPs for more than 10 years (OR 3.837, 95% CI 1.4–10.5) [27].

While the mechanism by which estrogen-containing OCPs may lead to hypothyroidism requires further investigation, one proposed mechanism could explain the findings of our study. Elevated estrogen levels increase TBG levels, possibly due to increased sialylation of the protein in the liver, which reduces its clearance rate [28]. A published review on thyroid function during pregnancy theorized that an increase in TBG could lead to a transient decrease in the free levels of T3 and, more significantly, T4, given the high-affinity TBG for T4 [29]. However, it has been reported that in iodine-sufficient pregnancies, a decrease in hormone levels is not significant [29]. Considering that both OCP use and pregnancy lead to increased estrogen levels, the proposed mechanism may be similar. However, this warrants further analysis.

Interestingly, our findings contrast with the literature, where higher SHBG levels have been observed in hyperthyroid patients [30]. For example, a study examining the relationship between hyperthyroidism and SHBG levels reported that 69% of the 82 patients sampled had elevated SHBG levels [31]. Additionally, SHBG has been used as a marker for thyroid hormone levels in clinical assessments [32]. Given that our results diverge from those of previous studies, our findings demonstrate the complexity of the interaction between SHBG and thyroid hormone levels, suggesting that the relationship between these variables may be bidirectional or context dependent. Further studies are needed to clarify the mechanisms of these interactions and how they may vary under different physiological conditions.

Our analysis also revealed that non-Qatari Arab participants presented an 8-fold greater odds of hypothyroidism than did Qatari and non-Arab participants. A cross-sectional study conducted in Qatar in 2021 reported a hypothyroidism incidence of 4.74%, with females accounting for 82.82% of the cases [6]. Additionally, a systematic review published in 2016 reported a greater prevalence of hypothyroidism in Libya (6.18%) than in Qatar [24]. These findings suggest that the increased odds of hypothyroidism among non-Qatari Arab individuals may be linked to the increased prevalence of this condition in certain Arab countries. However, the specific reasons for this increased risk, particularly among OCP users, require further investigation.

Although we did not observe a significant association between T2DM and hypothyroidism, previous research in Qatar involving 1000 participants revealed a higher prevalence of subclinical hypothyroidism in T2DM patients (4.6%) than in the general population (2.8%) [33]. The study also reported that subclinical hypothyroidism was associated with 2.82-fold increased odds of developing T2DM.

This study is the first to explore the associations among OCP use, SHBG levels, and hypothyroidism within the Qatari population. Despite the high prevalence of hypothyroidism and the widespread use of OCPs in the region, this topic has remained underexplored. One of the key strengths of our study is the large sample size, which provided sufficient power with good evidence against the null hypothesis regarding the association between OCP use and hypothyroidism. We also adjusted for critical covariates and potential confounders via multivariable logistic regression, enhancing the reliability of our findings.

Nevertheless, our study utilized data from the QBB, which presents certain limitations. The data collection method of the biobank poses a challenge in establishing a temporal relationship between the exposure and outcome. This constraint limits our ability to draw strong causal inferences. Furthermore, the lack of data on the type, duration, and dosage of OCP use restricts our capability to explore potential dose-response relationships comprehensively, and thus, restricts our prediction model. Additionally, discerning whether cumulative effects develop over time or whether specific dosage levels are more strongly associated with hypothyroidism becomes unattainable. Moreover, we acknowledge that the imbalance in group sizes is a limitation of our study. However, we addressed this issue rigorously by using multinomial logistic regression, which is a robust statistical method that accounts for sample size disparities while adjusting for key confounders. Nevertheless, future studies with a more balanced sample distribution would be ideal to strengthen the generalizability of the findings. We recommend that prospective cohort studies be developed to monitor and record detailed information on OCP, including but not limited to specific types, dosages, and durations of use. By collecting this data, such studies could also follow up and analyze changes in thyroid function indicators at multiple, predefined time points. This approach would allow for the evaluation of temporal relationships between OCP use and thyroid function more effectively, facilitating the identification of potential dose-response patterns and cumulative effects. Furthermore, the longitudinal design of these studies would allow for a more robust assessment of the causal relationship between OCP use and the risk of hypothyroidism. This method would provide a clearer understanding of the dynamics at play and contribute significantly to evidence-based recommendations for OCP usage and thyroid health monitoring.

## 5. Conclusion

In conclusion, our study revealed a significant association between hypothyroidism and SHBG levels above the 99th percentile, which is an indicator of estrogen-containing OCP use. We also found that compared with other Arabs and non-Arabs, Qataris are less likely to develop hypothyroidism when OCPs are used. In addition, we found no associations between specific risk factors, such as T2DM and hypothyroidism. Future research should investigate other potential risk factors that may influence the risk of hypothyroidism and explore the mechanisms by which OCP use could lead to hypothyroidism.

## Figures and Tables

**Table 1:** Characteristics of the study sample.

Factor		No hypothyroidism	Hypothyroidism	*p* value
Sample size		967	34	

Age, median (IQR)		44.0 (35.0, 53.0)	45.0 (36.0, 58.0)	0.33

Type 2 diabetes mellitus	No diabetes	707 (73.1%)	24 (70.6%)	0.74
	Diabetes	260 (26.9%)	10 (29.4%)	

Smoking status	Smoker	24 (2.5%)	0 (0.0%)	0.36
	Nonsmoker	943 (97.5%)	33 (100.0%)	

Nationality	Qatari	913 (94.4%)	29 (87.9%)	0.066
	Non-Qatari, Arabs	50 (5.2%)	3 (9.1%)	
	Others	4 (0.4%)	1 (3.0%)	

BMI	< 25	119 (12.3%)	7 (21.2%)	0.31
	25–30	308 (31.9%)	9 (27.3%)	

Abbreviations: BMI, body mass index; IQR, interquartile range.

**Table 2:** Characteristics of the study sample on the basis of SHBG levels.

Factor	Level	SHBG < 99^th^ percentile	SHBG ≥ 99^th^ percentile	*p* value
Sample size		990	11	

Age, median (IQR)		44.0 (36.0, 53.0)	34.0 (29.0, 40.0)	0.002

Type 2 diabetes mellitus status	No diabetes	721 (72.8%)	10 (90.9%)	0.18
	Diabetes	269 (27.2%)	1 (9.1%)	

Hypothyroidism status	No hypothyroidism	959 (96.9%)	8 (72.7%)	< 0.001
	Hypothyroidism	31 (3.1%)	3 (27.3%)	

Smoking status	Nonsmoker	966 (97.6%)	10 (100.0%)	0.62
	Smoker	24 (2.4%)	0 (0.0%)	

Abbreviations: IQR, interquartile range; SHBG, sex hormone-binding globulin.

**Table 3:** Different predictors and odds of hypothyroidism.

Predictors	Odds ratio	*p* value	[95% confidence interval]
SHBG 2^nd^ quartile	1.31	0.612	0.46–3.72
SHBG 3^rd^ quartile	1.5	0.438	0.54–4.22
SHBG 4^th^ quartile	1.65	0.359	0.57–4.78
Age	1.018	0.303	0.98–1.05
Arab ethnicity⁣^∗^	8.06	0.071	0.84–77.54
Other ethnicity⁣^∗∗^	1.87	0.32	0.55–6.37
T2DM	0.95	0.904	0.4–2.25
Constant	0.011	≤ 0.01	0.002–0.63

Abbreviations: SHBG, sex hormone-binding globulin; St. Err, standard error; T2DM, type 2 diabetes mellitus.

⁣^∗^Arab ethnicity excludes Qataris.

⁣^∗∗^Other ethnicities include non-Arab and non-Qataris.

**Table 4:** Multinomial logistic regression⁣^∗^ for hypothyroidism.

Primary outcome	SHBG 90^th^ percentile		SHBG 95^th^ percentile		SHBG 99^th^ percentile	
	OR 95% CI	*p* value	OR 95% CI	*p* value	OR 95% CI	*p* value
Hypothyroidism	1.00 (0.29–3.50)	0.998	1.46 (0.33–6.54)	0.62	8.88 (1.71–46.2)	0.009

Abbreviations: 95% CI, 95% confidence interval; SHBG, sex hormone-binding globulin; St. Err, standard error; T2DM, type 2 diabetes mellitus.

⁣^∗^Adjusted for age, ethnicity, T2DM.

## Data Availability

The dataset supporting the conclusions of this article are not publicly available due to restrictions imposed by the Qatar Biobank (QBB). Access to QBB data requires approval from the Qatar Biobank Steering Committee and adherence to the QBB's terms and conditions for data usage. Interested researchers may apply for access to the data at the QBB website: https://www.qphi.org.qa.

## Supporting Information

Directed acyclic graph (DAG) illustrating the assumed causal relationship between oral contraceptive pill (OCP) use and hypothyroidism, with age, ethnicity, and type 2 diabetes as potential confounders.
