# Supplementary material for: The Association Between Estrogen-Containing Oral Contraceptive Pills and Hypothyroidism
Source: Int J Endocrinol. 2025 Mar 18;2025:5978558. doi: 10.1155/ije/5978558 (PMC11936525; doi:10.1155/ije/5978558)
Supplement: Supporting Information — Additional supporting information can be found online in the Supporting Information section. [file 5978558.f1.docx]

**Supplementary File**

**Directed Acyclic Graph**
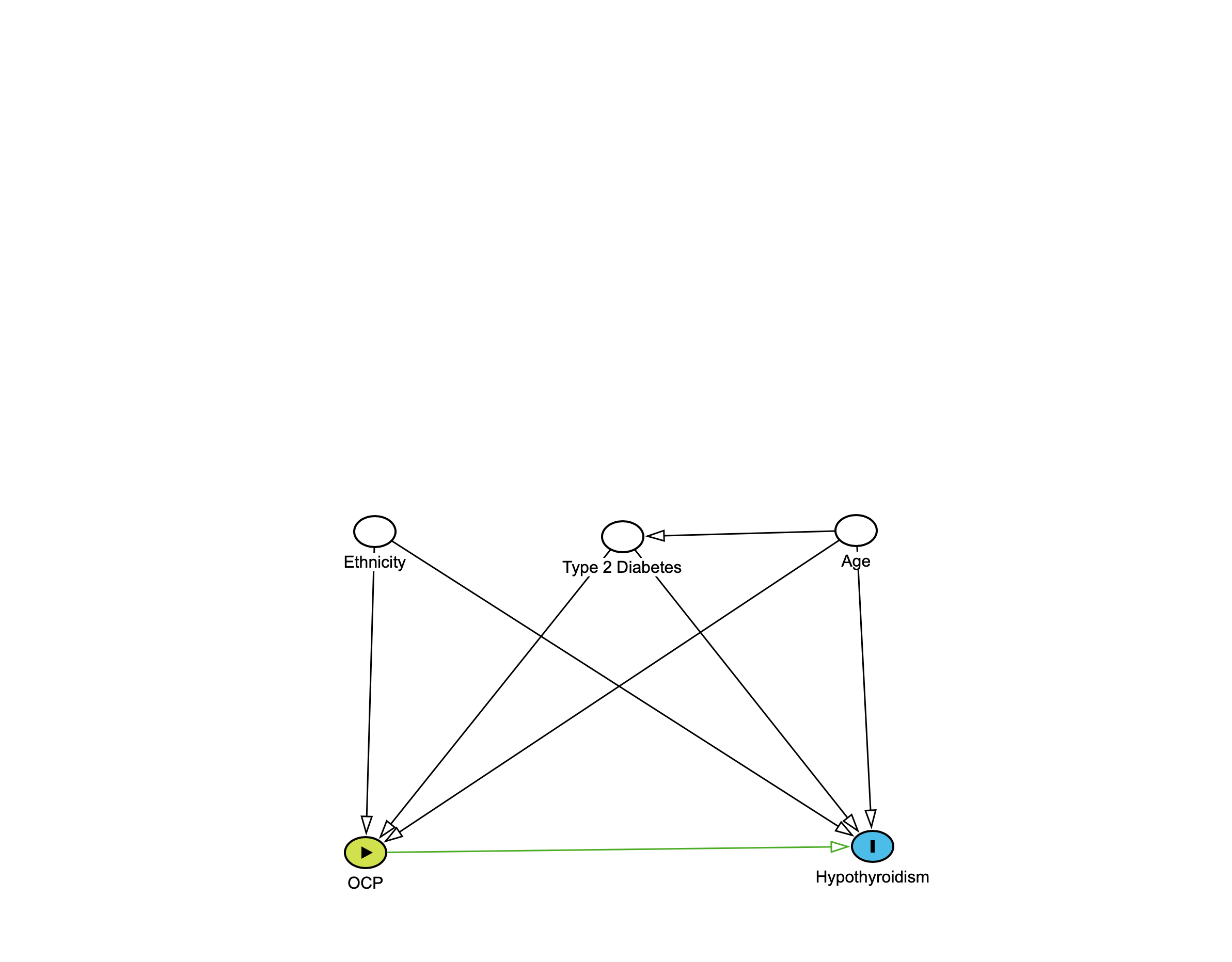


Directed acyclic graph (DAG) illustrating the assumed causal relationship between oral contraceptive pill (OCP) use and hypothyroidism, with age, ethnicity, and type 2 diabetes as potential confounders.
